# Impact of the Daily Use of a Microcrystal Hydroxyapatite Dentifrice on *De Novo* Plaque Formation and Clinical/Microbiological Parameters of Periodontal Health. A Randomized Trial

**DOI:** 10.1371/journal.pone.0160142

**Published:** 2016-07-28

**Authors:** Inga Harks, Yvonne Jockel-Schneider, Ulrich Schlagenhauf, Theodor W. May, Martina Gravemeier, Karola Prior, Gregor Petersilka, Benjamin Ehmke

**Affiliations:** 1 Department of Periodontology, University Hospital Muenster, Muenster, Germany; 2 Department of Periodontology, University Hospital Wuerzburg, Wuerzburg, Germany; 3 Society for Biometry and Psychometry, Bielefeld, Germany; 4 Private Practice, Wuerzburg, Germany; The Ohio State University, UNITED STATES

## Abstract

**Aim:**

This 12-week prospective, randomized, double-blind, two-center trial evaluated the impact of a microcrystalline zinc hydroxyapatite (mHA) dentifrice on plaque formation rate (PFR) in chronic periodontitis patients. We hypothesized that mHA precipitates cause delayed plaque development when compared to a fluoridated control (AmF/SnF_2_), and therefore would improve periodontal health.

**Material & Methods:**

At baseline and after 4 and 12 weeks, PFR and other clinical and microbiological parameters were recorded. Seventy periodontitis patients received a mHA or AmF/SnF_2_ dentifrice as daily oral care without hygiene instructions. Four weeks after baseline, participants received full mouth debridement and continued using the dentifrices for another 8 weeks.

**Results:**

Primary outcome PFR did not change statistically significantly from baseline to weeks 4 and 12, neither in mHA (n = 33; 51.7±17.2% vs. 48.5±16.65% vs. 48.4±19.9%) nor in AmF/SnF_2_-group (n = 34; 52.3±17.5% vs. 52.5±21.3% vs. 46.1±21.8%). Secondary clinical parameters such as plaque control record, gingival index, bleeding on probing, and pocket probing depth improved, but between-group differences were not statistically significant. Microbiological analyses showed similar slight decreases in colony-forming units in both groups.

**Conclusion:**

In patients with mild-to-moderate periodontitis, periodontal therapy and use of a mHA-or AmF/SnF_2_ dentifrice without instructions induced comparable improvements in periodontal health but did not significantly reduce the PFR.

**Trial Registration:**

ClincalTrials.gov NCT02697539

## Introduction

Efficient mechanical plaque control through meticulous oral home care employing different kinds of toothbrushes and dentifrices is the basis of most periodontal treatment regimens [1]. Commonly, dental plaque is disrupted supragingivally by the mechanical action of the toothbrush bristles and swept away from the dental surface. The cleaning efficacy is significantly enhanced by the use of toothpaste [2, 3]. In populations without access to customary oral hygiene devices, up to 95% of dental surfaces exhibit visible plaque [4]. In contrast, subjects living in industrialized countries and practicing regular oral hygiene have on average 40% to 60% of surfaces covered with plaque [5].

However, as well as the mechanical removal of plaque, pharmacological measures to inhibit its growth have also been a focus of interest in recent years [6, 7]. For decades fluoride compounds have been regarded as effective chemical agents, not only to prevent caries, but also to reduce dental plaque and therefore improve gingival health. The inclusion of fluorides in toothpastes is regarded as a gold standard in oral care today. Fluorides are widespread used and a wealth of scientific data suggests different dosages [8]. However, there is still a scientific interest in developing plaque reducing toothpastes with new antimicrobial modes of action.

Most established dentifrices improve cleaning efficacy due to containing abrasive components (such as hydrated silica) and furthermore, they reduce recolonization through the addition of agents with antimicrobial properties, like stannous fluoride. In recent years, new specifications for biomimetically synthesized hydroxyapatites in oral hygiene have led to a revival of a research topic pioneered by Hüttemann and Dönges in 1987 [9]. In addition to several in vitro studies, a recently published clinical trial demonstrated the benefits of incorporating zinc hydroxyapatite into oral care products; use of a zinc hydroxyapatite-containing toothpaste resulted in the formation of a hydroxyapatite-rich coating, strengthening the enamel surface [10]. In another study, daily use of a zinc hydroxyapatite-containing toothpaste significantly reduced dentinal hypersensitivity after four and eight weeks [11]. The working principle is largely based on the application of highly concentrated biomimetic zinc hydroxyapatite. Due to their physicochemical properties microcrystalline zinc hydroxyapatite (mHA) particles are adhesive and durable on bacterial surfaces. This may create various therapeutic options including inhibition of the development of dental biofilms. In vitro, microcrystalline zinc hydroxyapatite in dentifrices coats the surfaces of oral bacteria during tooth brushing and may thus reduce their ability to aggregate or co-aggregate on intraoral surfaces [12]. As a result this should lead to reduced de novo plaque formation in vivo and, in patients suffering from gingivitis or periodontitis, to an improvement of gingival health. In periodontitis or gingivitis patients, toothpaste which reduces or delays bacterial growth may support treatment outcomes favorably.

This trial aims to test the hypothesis that the daily unsupervised and uninstructed use of a dentifrice containing microcrystalline zinc hydroxyapatite by patients suffering from periodontal disease will result in a significantly more pronounced reduction of de novo plaque formation than the daily use of a control dentifrice containing conventional abrasives and aminefluoride and stannous fluoride as antimicrobial active agents.

## Materials and Methods

The investigation was designed as a double-blind, parallel-group, randomized, two-center trial (Dept. of Periodontology, University Hospital, Muenster, Germany and Dept. of Periodontology, University Hospital, Wuerzburg, Germany) over a 12-week period. The primary outcome parameter was the observed difference in de novo plaque formation as recorded by the plaque formation rate (PFR) [13], between subjects using the carbonate/hydroxyapatite dentifrice (test: mHA, BioRepair, Wolff, Bielefeld, Germany) and those using a dentifrice containing an aminefluoride/stannous fluoride (control: AmF/SnF_2_, Meridol, GABA, Lörrach, Germany). Patient recruitment started in March 2011 and finished in September 2011. The follow-up period was from April 2011 through February 2012. The study database was closed in August 2012.

**Fig 1:**
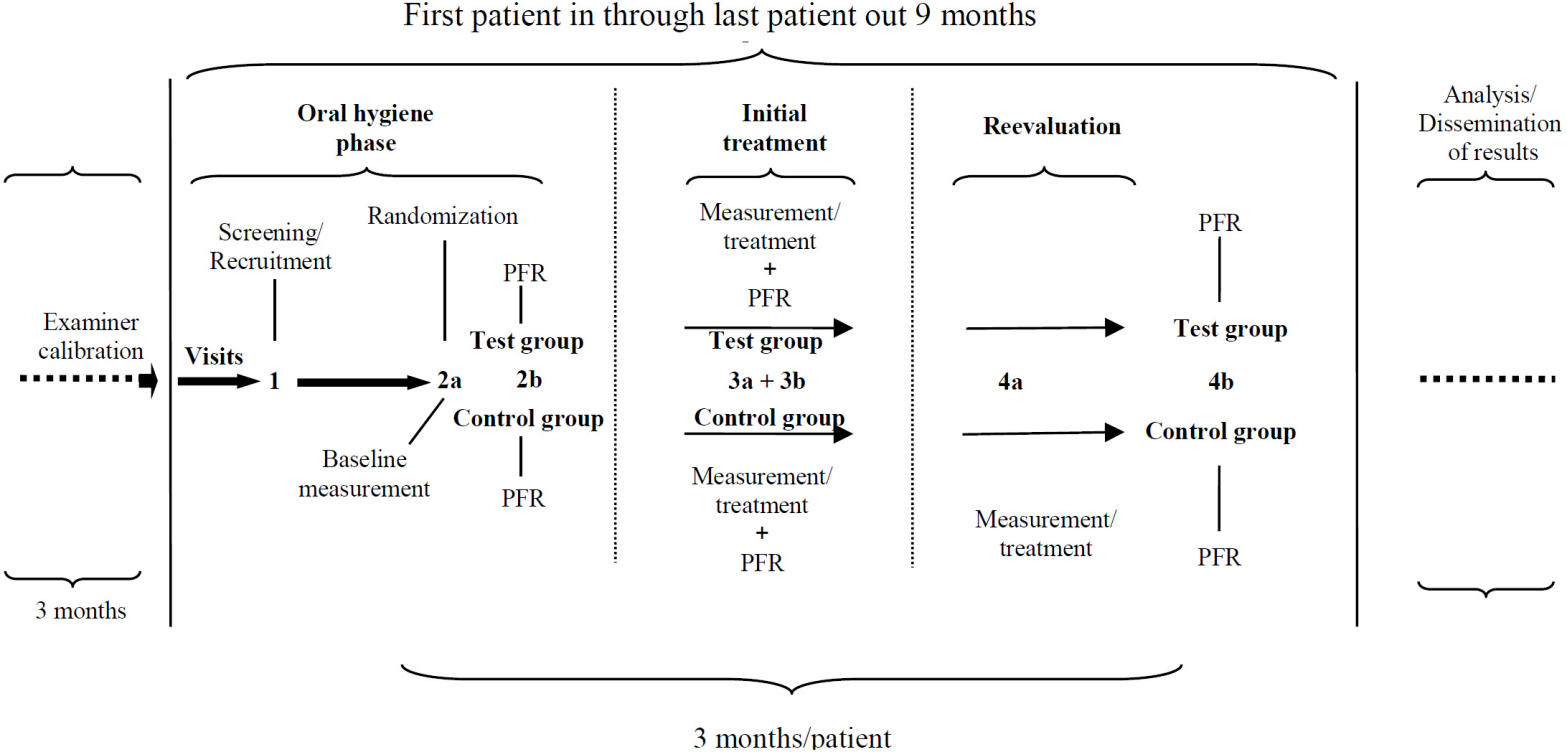
Study design. Visits 2b, 3b, and 4b were scheduled 24 hours following visit 2a, 3a, and 4a, respectively. PFR: Plaque formation rate; Test: microcrystalline zinc hydroxyapatite (mHA) dentifrice; Control: fluoridated control (AmF/SnF_2_) dentifrice.

Patients were screened at visit 1 and inclusion/exclusion criteria were verified. The study design is schematically depicted in Fig 1. This design allows to evaluate the clinical effect of supragingival debridement and supra- and subgingival debridement with adjunctive use of one of both toothpastes.

### Study subjects

Patients suffering from untreated localized mild-to-moderate chronic periodontitis were initially included into the study [14].

### Inclusion criteria

- Pocket probing depths (PPDs) of ≥ 4 mm at a minimum of 4 teeth (except third molars)
- age ≥ 18 to ≤ 75 years
- at least 10 natural teeth in situ (except third molars)
- non-smokers

### Exclusion criteria

- known systemic diseases that may influence the periodontal conditions
- regular consumption of drugs that may interfere with periodontal conditions
- undergoing or requiring extensive dental or orthodontic treatment
- pregnancy or breastfeeding
- professional periodontal therapy during the 6 months prior to baseline
- periodontal pockets ≥ 6 mm in more than 2 sextants

### Examiner calibration and randomization

Prior to the recruiting phase, all examiners (qualified dentists) were calibrated for measuring the PFR index [15]. At recruiting visit 1, patients were screened for eligibility and invited to take part in the study. At visit 2a eligible patients who had given their written informed consent for participation were randomly assigned to either the test or control group. Participants were randomly assigned to the test or the control group by the use of computerized center-specific randomization lists. Quad-block randomization lists were generated for each center by a statistician who was not involved in any other aspect of the trial. Randomization lists were stored exclusively at the study centers. Randomization was performed by 2 study nurses who were not involved in measurements or treatment of the participating patients.

### Clinical measurements

The primary outcome of PFR was measured 24 hours after all teeth were treated mechanically (visits 2b, 3b, and 4b, respectively). Secondary outcomes, full mouth plaque control record (PCR, [16]), gingival index (GI, [17]), pocket probing depths (PPD), bleeding on probing (BOP, [18]), gingival recession (GR), and microbiological samples were recorded at baseline (visit 2a), after 4 weeks (visit 3a) and after 12 weeks (visit 4a). Chronology of all measurements is shown in Table 1.

**Table 1:** Chronology of study visits, examinations, and therapy.

		Oral hygienePhase		Treatment		Reevaluation	
	Recruitment	Baseline		Treatment*		Reev**	
Visit	1	2a	2b***	3a	3b***	4a	4b***
**Recruitment**							
Periodontal Screening	X						
Medical Health History	X	X		X		X	
Inclusion/exclusion criteria	X						
Study information	X						
Informed consent	X						
Registration	X						
Randomization/balancing		X					
**Treatment activities**							
Oral hygiene instructions		X	X	X	X	X	
Supragingival debridement		X	X	X	X	X	X
Subgingival debridement				X	X		
Dentifrice dispense/return			X				X
**Examinations**							
Plaque formation rate index			X		X		X
Plaque index		X		X		X	
Gingival index		X		X		X	
Gingival recession		X		X		X	
Bleeding on probing		X		X		X	
Pocket probing depth		X		X		X	
**Microbial samples**							
Subgingival samples		X		X		X	
Supragingival samples		X		X		X	
Interproximal samples		X		X		X	
**Smoking**							
CO measurements	X	X		X		X	

* Within approx. 4 weeks after baseline

** Two months after treatment, ± 14 days

*** Visits a + b within 24 hours.

All measurements were recorded at 6 sites per tooth. A standard Florida Probe hand-piece was used for measurements (Florida Probe Corp., Gainsville, FL, USA). Non-smoking status was monitored by measuring carbon monoxide concentration in exhaled air (≤ 3 ppm CO; Bedfont-Smokerlyzer, Bedfont, UK; visits 2 through 4). For the recording of PFR and PCR, all teeth were stained with a plaque revealer (Mira 2-Ton Miradent, Hager & Werken GmbH, Duisburg, Germany).

### Periodontal therapy

Full mouth supragingival cleaning was performed after assessment of secondary parameters, but 24 hours prior to the assessment of the PFR scores. This was done with sonic scalers with micro tips (Sonicflex 2003L, KAVO, Bieberach, Germany) and subsequent air polishing with glycine powder (ClinPro, EMS, 3M Espe, Seefeld, Germany) and an air flow device (EMS S1, EMS Electro Medical Systems, Nyon, Switzerland) at visits 2a, 3a, and 4a. Subsequently after “a” visits, patients were instructed to refrain from any oral hygiene measures for the following 24 hours. After each PFR assessment, patients were instructed to resume their regular oral hygiene. Dentifrices for test and control groups were dispensed after visit 2b.

Additionally, periodontal therapy performed at visit 3a comprised full mouth supragingival and, where indicated, subgingival debridement (sites with PPD ≤ 4mm). Subgingival debridement was performed under local anesthesia using sonic scalers followed by air polishing with glycine powder. The period of time between visits was chosen to evaluate the effects of the different mechanical approaches in conjunction with the different toothpastes (visit 2a to visit 3a: 4 weeks after the supragingival debridement and oral hygiene, and visit 3a to 4a: 8 weeks after the supra- and subgingival approach).

### Microbiological sampling

**Fig 2:**
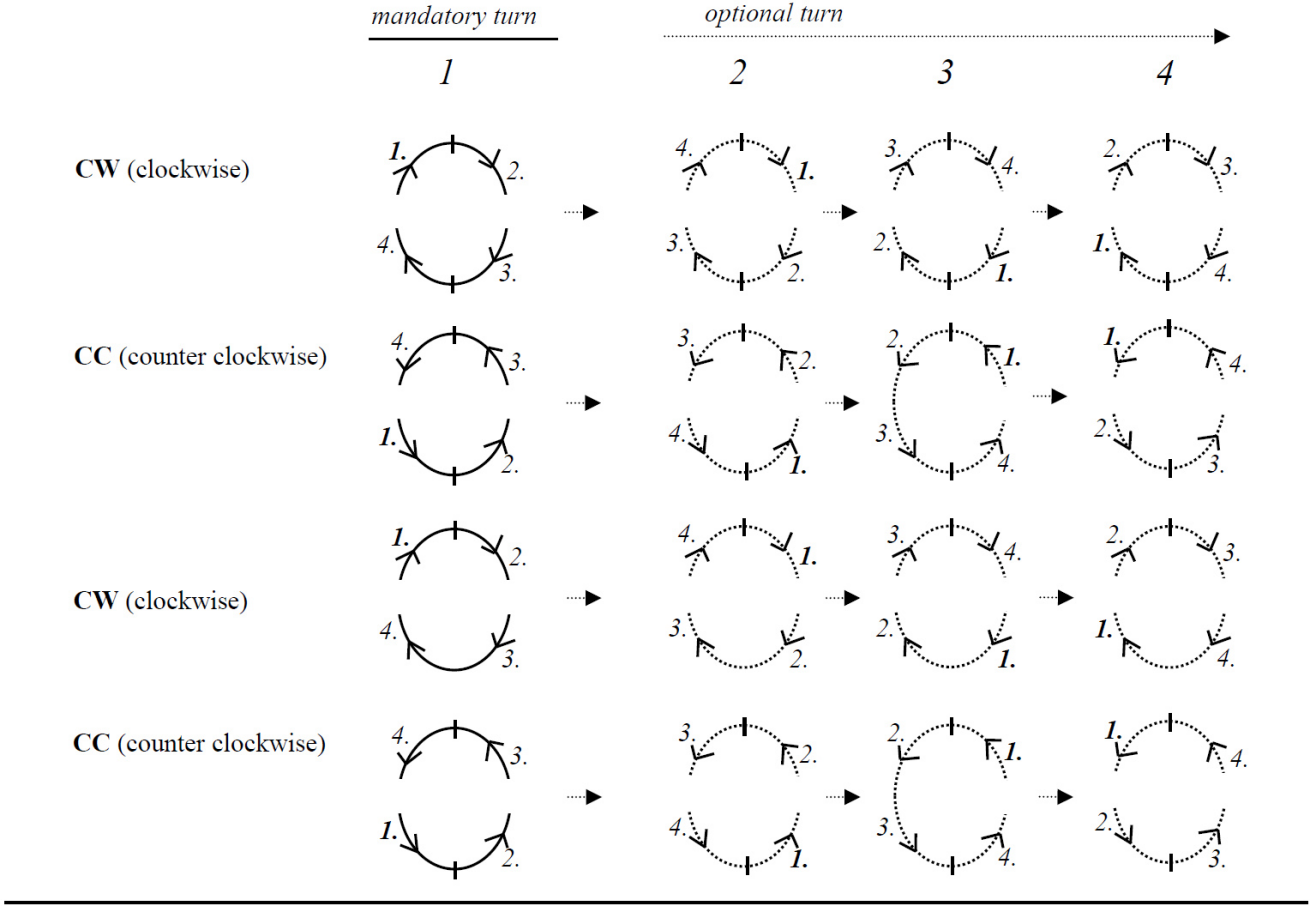
Sample tooth selection. Selection process: According to the randomization list, sample teeth are selected in a clockwise (CW) or counter clockwise (CC) direction. Example: For clockwise selection, start at the upper right quadrant from the most distal tooth going towards the mesial (CW, mandatory turn 1)
Select the most distal tooth in this quadrant that meets the criteria (at least 1 site with at least 1 site with PPDs of ≥ 4 mm); if 1 tooth is selected or none meets the criteria thencontinue clockwise in the upper left quadrant and now select the most mesial tooth that meets the inclusion criteria, if 1 tooth is selected or none meets the criteria,continue clockwise in the lower left quadrant and select the most distal tooth that meets the inclusion criteria, if 1 tooth is selected or none meets the criteria,continue clockwise in the lower right quadrant and select the most mesial tooth that meets the inclusion criteria, stop if 1 tooth in each quadrant has been selected, if less than 4 teeth have been selected,continue clockwise (optional turn 2), and start in the upper left quadrant going from mesial towards distal,continue clockwise with optional turns 3 and 4 until a total number of 4 sample teeth have been included. For counter clockwise proceed as indicated in the figure above.

For microbiological analysis, samples were taken at visit 2a from 4 randomly selected teeth which were subsequently resampled at visits 3a and 4a. Sampling strategy considered equal distribution of single- and multi-rooted teeth in the upper and lower jaw (Fig 2). The same kind of sterile paper points (ISO 45, Roeko, Ulm Germany) were used for sampling at supragingival buccal/lingual sites, at supragingival interproximal sites, and subgingival sites, and were inserted for 10 seconds at each site. Supragingival plaque was collected from 4 sites per sample tooth. Buccal and lingual site samples were taken from the area close to the gingival margin. Interproximal supragingival plaque samples were collected by inserting a sterile paper point horizontally in the buccal-lingual direction near the gingival margin. The 2 samples from buccal/lingual sites as well as the 2 samples from interproximal sites were pooled, resulting in 2 pooled samples from each subject per visit 2a, 3a, and 4a. Additionally, 2 subgingival plaque samples were taken from periodontal pockets using sterile paper points and subsequently pooled for each subject.

All plaque samples were collected in transport tubes containing 500 μl Ringer-Glycerin-Solution, and were stored in liquid nitrogen until further analysis. Whole bacterial counts were quantified by culture at visits 2a, 3a, and 4a.

### Microbiological analysis

Directly after sampling paperpoints were given in tubes with ringer glycerin and these samples were stored in liquid nitrogen. To obtain equal conditions for the samples of both participating centers, samples from center 2 are shipped to Muenster on dry ice (-80°C) every second month and are placed back in liquid nitrogen directly upon receipt in the microbiological laboratory. Analysis of all microbiological samples started 2 month after the first sampling, when the first shipment of the external center arrived.

The pooled samples were sonicated for 10 seconds (Sonotex RK 82, Bandelin Electronic AG, Berlin, Germany) and diluted in tenfold steps. The undiluted suspension (0.1ml) and aliquots of the dilutions (0.1 ml) were spread on different culture media. In parallel supragingival plaque samples were spread on CDC agar and were incubated in an aerobic atmosphere at 37°C overnight. For quantitative enumeration the undiluted and diluted suspensions were spread on non-selective blood agar (CDC agar) plates containing 5% defibrinated sheep blood supplemented with 5mg/l hemin (Merck, Darmstadt, Germany), 1 mg/l vitamin K_1_, and 10 mg/l N-acetylmuramine acid (NAM) [19]. The plates were incubated in an atmosphere containing N_2_ (85%), H_2_ (10%), and CO_2_ (5%) for 7 days. Total cultivable counts were assessed for each of the plaque samples. The evaluation of pooled plaque samples on CDC agar and the total cultivable counts were reported quantitatively as colony-forming units (CFU/ml).

### Statistical analysis

Using data based from a non-blinded pilot approach, the confirmatory objective was to detect a clinically relevant difference of 13% between the groups (15% mHA, 2% AmF/SnF_2_) in PFR with standard deviation SD = 11%, type-I error rate α = 0.05 and power 1-β = 0.8. In total, 20 evaluable patients per group were required. Charts for the power function for analysis of variance tests were applied and a one sided-alpha at the 0.05 level was used in the power calculation [20]. The hypothesis was that the use of mHA toothpaste will reduce the PFR about 15% compared to 2% with AmF/SnF_2_ toothpaste. However, the authors were doubtful concerning the acceptance of the mHA toothpaste by the participants because this was the first clinical trial with this toothpaste. Therefore, recruitment was increased to account for a higher dropout rate.

Data were presented as means ± standard Deviation (SD). The primary efficacy endpoint was PFR at visit 3 (after 4 weeks of treatment). P-values ≤ 0.05 were considered as statistically significant. Main secondary efficacy endpoints were PI, GI, BOP, PPD, and GR at visit 3 (after 4 weeks of treatment) as well as OHIP-G 49 and CSQ-8 at visit 4. The attachment level was calculated from measurements of pocket probing depth and gingival recession. For both primary and secondary outcome parameters, analysis of covariance (ANCOVA) was used to test the difference between groups. ANCOVA was performed using ‘group’ (control or test group) and ‘center’ (Muenster or Wuerzburg) as fixed effects, and the baseline value (Visit 2) of the corresponding endpoint as covariate. In addition, analyses of variance (ANOVA) with repeated measures (V2, V3, and V4) were performed for the primary and secondary endpoints unless otherwise stated.

For statistical analyses of microbiological data, CFU were log-transformed and log10 reduction factors (RF V2/V3 = log (CFU V2)-log (CFU V3), RF V3/V4 = log (CFU V3)-log (CFU V4) were calculated. These data were analyzed by non-parametric tests (Mann-Whitney Test for between-group comparisons, the Wilcoxon signed rank test was used for within-group comparisons). Reported p-values for secondary endpoints were not adjusted for multiple comparisons (explorative analysis).

The IBM SPSS Statistics software for Windows (IBM Corp., Armonk, NY,USA) was used for statistical analyses.

### Ethical approval

All procedures performed in this study involving human participants were in accordance with the ethical standards of the institutional and/or national research committee and with the 1964 Helsinki declaration and its later amendments or comparable ethical standards. Study protocol and informed consent were approved by the ethics committee of the medical council of Unterfranken, Germany (Ref. No. 2/11, date: Febr. 1^st^ 2011, S1 File–PFR Protocol 203011). Informed consent was obtained from all individual participants included in the study. Initially the authors did not register the trial because the used toothpastes were cosmetics and no drugs; however, the trial was registered subsequently at www.clinicaltrials.gov (ClinicalTrials.gov NCT 02697539).

## Results

**Fig 3:**
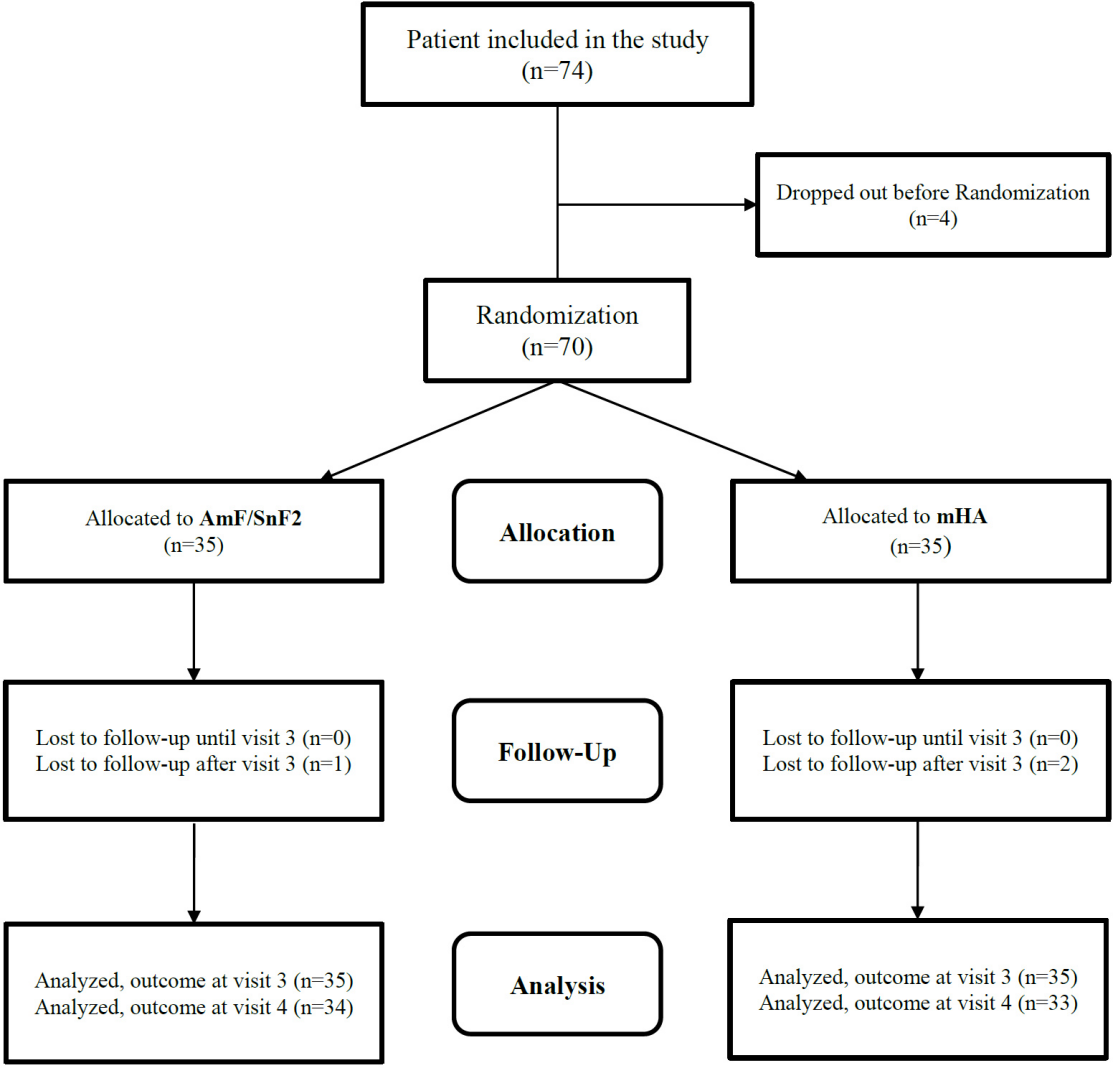
Patient flow chart (mHA: microcrystalline zinc hydroxyapatite dentifrice, AmF/SnF_2_: aminefluoride/stannous fluoride).

Out of a total of 74 recruited patients, 70 were randomized, and 67 completed visits 2 through 4 (Fig 3).

Mean age of the patients was 52.8±13.2 in the mHA and 53.5±12.7 years in the AmF/SnF_2_ group. In the mHA group, 51.4% of patients were female and 57.1% were female in the AmF/SnF_2_ group. At baseline, mean numbers of missing teeth (without wisdom teeth), fillings, and crowns were in mHA 1.5±1.6, 8.8±5.4, and 3.9±4.0, respectively; and 1.5±2.0, 8.1±5.0, and 5.4±4.2 in the AmF/SnF_2_ group, respectively. All included patients had CO concentrations in exhaled air of 3 ppm or less, only 1 patient showed at visit 4a 5 ppm CO in exhaled air. This patient was not excluded from analyses.

### Clinical variables

#### Changes from visit 2 through visit 3

The primary outcome variable of PFR scores varied slightly but not significantly in both the mHA (p = 0.442) or in the AmF/SnF_2_ group (p = 0.755), and no significant intergroup differences were detected (ANCOVA, p = 0.473; 95% confidence interval (CI95) = [-4.8, 10.3]; Table 2).

**Table 2:** Clinical parameters at baseline and follow ups (means±SD.

	Baseline (visit 2)		4 weeks (visit 3)		12 weeks (visit4)	
	Test	Control	Test	Control	Test	Control
PFR (%)	51.7±17.2	52.3±17.5	48.5±16.6	52.5±21.3	48.4±19.9	46.1±21.8
PCR (%)	61.5±17.9	64.3±19.6	53.7±17.2	52.7±18.6	51.3±18.6	48.7±19.4
GI	1.1±0.4	1.1±0.5	0.8±0.4	0.8±0.4	0.6±0.3	0.7±0.4
BOP (%)	22.8±21.5	20.5±16.9	16.0±17.3	15.7±12.8	14.4±13.9	13.5±11.9
PPD (mm)	2.6±0.4	2.5±0.3	2.5±0.5	2.4±0.4	2.2±0.4	2.3±0.4
PPD (%)						
≤ 3.4mm	85.4±12.3	85.4±8.7	87.6±13.2	90.3±7.3	92.6±7.7	92.2±7.3
3.5–6.4mm	14.2±11.7	14.3±7.7	11.9±12.4	9.4±7.1	7.3±7.5	7.6±7.1
≥ 6.5mm	0.4±1.2	0.3±0.6	0.5±1.6	0.3±0.8	0.2±0.5	0.3±0.7
GR (mm)	0.5±0.6	0.3±0.3	0.5±0.6	0.4±0.4	0.5±0.5	0.3±0.3
AL (mm)	3.1±0.8	2.8±0.4	2.9±1.0	2.8±0.5	2.7±0.8	2.6±0.5

Changes of the clinical parameters from visit 2 (baseline) through visit 3 (after 4 weeks of brushing) and visit 4 (3 months after continuous brushing and professional therapy). ANCOVA did not confirm any significant differences between groups in the clinical parameters at visit V3 (p>0.20). In addition, ANOVA (V2, V3, V4) showed no significant group x time interaction, whereas the time-effect was noticeable (p<0.001) for all of the clinical parameters, except PFR (p = 0.069).

PFR: Plaque formation rate, PI: O’Leary Plaque index, GI: Löe & Silness Gingival Index, PPD: Pocket probing depth, GR: Gingival recession, AL: Attachment level. Means ± standard deviation.

As a secondary outcome parameter from visit 2 through visit 3, PCR scores decreased noticeably in both groups (ANOVA, time effect p < 0.001). However, between group differences were not noticeable at visit V3 (ANCOVA, p = 0.491, CI95 = [-9.1, 4.4]). Similarly, BOP and GI scores were significantly decreased in both groups over the course of the study (ANOVA: time effect p < 0.001, respectively), but the differences between the test and control groups at visit 3 were not noticeable (ANCOVA, p = 0.779 CI95 = [-3.95, 5.25] and p = 0.563 CI95 = [-0.11, 0.20], respectively; Table 2). There were no significant changes in PPD either within the groups from visit 2 through 3, or between mHA and AmF/SnF_2_ groups (ANCOVA, all p = 0.510, with CI95 = [-0.173, 0.087]. There were no noticeable between- or within-group differences regarding the attachment level at visit 3 (Table 2).

#### Changes from visit 2 through visit 4

PFR was not significantly different between the mHA and AmF/SnF_2_ groups from visit 2 through 4 (ANOVA, p = 0.436 for group x time interaction, Table 2). However, there was a noticeable center effect. The mean PFR increased slightly in the MuensterMuenster center from visit 2 through 4 in the test (51.7 ± 17.8% to 59.2 ± 16.7%) and control group (57.6 ± 18.7% to 62.4 ± 15.0%), whereas in the WuerzburgWuerzburg center mean PFRs decreased in both groups (51.7 ± 17.0% to 35.6 ± 15.6% and 46.4 ± 14.5% to 27.7 ± 10.5%, respectively). However the observed differences between the mHA and AmF/SnF_2_ groups in either center were not significant.

Secondary outcome variables are shown in Table 2. From visit 2 through visit 4, PCR scores significantly decreased in both groups (ANOVA, time effect p < 0.001). However, PCR scores for the mHA and AmF/SnF_2_ groups did not differ significantly during the course of the study (ANOVA, p = 0.512 for group x time interaction). BOP and GI scores decreased significantly in both groups over the course of the study (ANOVA: time effect p < 0.001 for each parameter mentioned above), but the differences between the mHA and AmF/SnF_2_ groups over the course of the study (ANOVA, group x time interaction, p = 0.803 and p = 0.688, respectively) were not significant.

Reduction of PPD was significant within the groups from visit 2 through 4 (ANOVA, p < 0.001), but change between mHA and AmF/SnF_2_ groups were not significant over the course of the study (visit 2 through 4, ANOVA, p = 0.230). There were no significant between- or within-group differences regarding attachment level over the course of the study (Table 2). Finally, no new caries lesions were detected in both, mHA and AmF/SnF_2_ groups, over the study period.

### Microbiological results

At the supragingival buccal and lingual sites a slight decrease in colony-forming units was found from visit 2 through 4, using aerobic and anaerobic cultivation. CFUs from the subgingival area remained unchanged between visits 2 to 3 and decreased from visit 3 to 4 similarly in both mHA and AmF/SnF_2_ groups. In contrast, the CFUs at supragingival interproximal sites did not change over the course of the study in either aerobic or anaerobic cultivation (Table 3).

**Table 3:** Microbiological results at baseline and follow ups (means±SD). Log–transformed bacteria counts.

	Baseline		4 weeks		12 weeks	
	Test	Control	Test	Control	Test	Control
**Supragingival**						
*Buccal and lingual sites*						
Aerobic	5.3±1.2	5.4±0.9	5.0±0.9	5.4±0.8	5.0±1.0	4.9±1.0
anaerobic	5.7±1.2	5.8±1.2	5.4±1.2	5.8±0.9	5.3±1.1	5.3±1.3
*interproximal sites*						
Aerobic	6.5±0.9	6.5±0.6	6.5±0.6	6.7±0.7	6.4±0.7	6.5±0.6
Anaerobic	7.2±0.9	7.3±0.6	7.3±0.7	7.4±0.6	7.1±0.6	7.2±0.6
**Subgingival**						
Anaerobic	6.4±1.2	6.8±0.6	6.5±1.0	6.8±0.6	6.3±0.9	6.4±0.7

Log-transformed bacteria count at visit 2 (baseline), visit 3 (after 4 weeks of brushing) and visit 4 (3 months after continuous brushing and professional therapy). Means ± SD, standard deviation.

The microbiological analysis did not result in differences concerning reduction of CFU in either the supragingival area (buccal, lingual or interproximal) or in the subgingival area for both groups from visit 2 to 3 (Table 4). However, at buccal and lingual sites, the reduction of CFUs after aerobic culture was more pronounced in mHA group patients (p = 0.051) from visit 2 to 3, whereas the reduction from visit 3 to 4 was noticeably greater in the AmF/SnF_2_ group (p = 0.016).

**Table 4:** Microbiological results from baseline through follow ups (means±SD). Log-transformed reduction factors.

	Baseline through 4 weeks		4 weeks through 12 weeks	
	Test	Control	Test	Control
**Supragingival**				
*Buccal and lingual sites*				
Aerobic	0.3±1.0	-0.1±0.7	-0.1±0.7	0.5±1.0
Anaerobic	0.3±1.1	0.0±1.1	0.2±1.1	0.5±1.1
*interproximal sites*				
Aerobic	-0.0±0.7	-0.2±0.7	0.1±0.5	0.2±0.8
Anaerobic	-0.1±0.6	-0.1±0.6	0.2±0.5	0.2±0.5
**Subgingival**				
Anaerobic	-0.1±1.2	0.0±0.7	0.2±1.0	0.4±0.7

Log-transformed reduction factors. For supragingival buccal and lingual sites in the visit 2 through visit 3 period, aerobe cultivation resulted in more pronounced reduction of bacterial counts in the test group compared to the control group (p = 0.051), whereas between visit 3 and visit 4, the reduction in the control group was significantly greater compared to the test group (p = 0.016). Differences between groups for supragingival anerobic, interproximal and subgingival reduction factors were insignificant.

## Discussion

The present trial evaluated the effect of two dentifrices which differed in their composition as regards to the type of abrasives used and the inclusion of fluorides. Both ingredients have some impact on clinical and microbiological parameters of periodontal health. The data analysis revealed comparable, beneficial effects in the parameters of periodontal health evaluated in subjects affected by mild-to-moderate chronic periodontitis. However, caries prevention capacities of the mHA toothpaste was not an outcome parameter of this trial, but no new caries lesions were detected in both groups over the course of the study. Because this trial did not recruited selectively patients with risk for caries, new clinical studies using mHA toothpastes should be designed to evaluated this topic.

The addition of triclosan, chlorhexidine, fluorides or metal ions in dentifrices primarily aims to enhance the biocidal effect within oral microflora. There is extensive published data on the beneficial impact of dentifrices and mouthwashes containing aminefluoride (AmF) and stannous fluoride (SnF_2_) on periodontal health. In general, dentifrices containing AmF/SnF_2_ improved gingival health, but showed inconsistent effect on dental plaque indices [2]. Likewise in the present study, oral hygiene and professional therapy in conjunction with zinc carbonate/hydroxyapatite dentifrice also led to improved periodontal health and better plaque control but not to decreased de novo plaque formation.

The patients involved in the trial already practiced satisfactory though not perfect oral hygiene routines. Possibly, recruiting patients with initially high plaque and gingival scores would have resulted in somewhat more distinct effect of dentifrices and periodontal therapy. Recruiting a “real life” patient sample with satisfactory oral hygiene levels and mild-to-moderate chronic periodontitis, effects of the dentifrices must result in less pronounced changes in parameters as plaque and gingival scores. However, daily oral hygiene is, besides others, a relevant strategy to improve oral health, therefore, also little improvements caused by the simple use of 1 of both dentifrices may be of clinical relevance on the long run.

The present study design comprises two parts. In part 1, i.e. visit 2 through visit 3, the blinded dentifrices were given to the study patients without any additional professional periodontal therapy or behavior changing oral hygiene instructions. Thus, it was possible to estimate the effect of both products on oral health in an uninstructed “over the counter” model. In part 2, from visit 3 through 4, efficacious professional periodontal therapy was added. Except for the PFR index all clinical parameters in both groups improved from visits 2 through 3 and further from 2 through 4. Data analysis however failed to detect any significant differences between both test groups. In this clinical trial, PCR scores as well as GI scores decreased significantly irrespective of the antimicrobial ingredients (AmF/SnF_2_ or zinc carbonate/hydroxyapatite) of the dentifrice used by the study participants.

The zinc carbonate/hydroxyapatite dentifrice evaluated follows a more holistic approach to improve oral health. This comprehensive approach is based on the moderate biocidal activity of zinc ions combined with the activity of zinc hydroxyapatite. Exposing *Streptococcus mutans* cultures to microcrystalline zinc hydoxyapatite rinsing solution of different concentrations in vitro resulted in reduced bacterial viability. This effect was also seen at higher dilutions. In controls which rinsed with chlorhexidine, the biocidal effect was more dose-dependent [12]. Further in situ experiments with zinc carbonate/hydroxyapatite solutions revealed a significant reduction of oral bacteria adherence to enamel surfaces. The reduction of bacterial adherence observed was comparable to the reduction associated with the application of chlorhexidine [12].

Using different sample locations for supragingival plaque samples, we tried to distinguish toothpaste-related effects on periodontal parameters at sites with direct contact to the bristle tips (buccal and lingual) and those which were not accessible for the bristle tips (interproximal). Supragingival self-care oral hygiene with toothbrush and dentifrice alone (visit 2 to visit 3) did not result in a reduction of either the buccal, lingual or the interproximal supragingival bacterial count. The reduction of supra- and subgingival bacterial counts was only significant within the groups after combined supra- and subgingival debridement had been performed (visit 3 to visit 4). However changes in bacterial counts observed at the interproximal sites also failed to reach significance after professional debridement (visit 3 to visit 4). The main effect of improving clinical parameters in the present trial should be attributed to the professional therapy between visit 3 and 4. However, the significant decrease of PCR and GI scores, despite the limited reduction of total bacterial counts suggests an effect on microbial composition and diversity rather than total numbers. In a recent analysis, using a next-generation sequencing technique, we detected a pronounced tendency to species diversification in the dental biofilm after supra- and subgingival periodontal therapy [21]. This finding may help to explain the apparent contradiction of the improvement of plaque and gingival indices and the limited effect on bacterial count. However, analogous to the periodontal-therapy induced diversification of the bacterial community, the pharmacological mechanisms of the dentifrices may also possibly stimulate and enhance that diversification. Therefore, it seems more plausible that the short-term biocidal properties of both dentifrices are rather limited, but may exert beneficial microbial shifts in the composition of the dental biofilm associated with reduced gingival inflammation, thus possibly improving periodontal health in the long run.

It is common practice in most clinical tooth brushing studies to improve participants’ capabilities of daily oral hygiene by intensive oral hygiene training sessions prior to baseline. However, those training sessions may influence and bias the effects measured in the studies attributable to the investigational product. In this study therefore participants were asked to continue their usual oral hygiene habits without any further instructions or training sessions.

When interpreting the study results, the fact that we included patients (i) suffering from mainly mild-to-moderate chronic periodontitis, (ii) patients who already practiced satisfactory though not perfect oral hygiene care, and (iii) the patients whose oral hygiene habits were not intentionally altered, must be taken into account. In contrast to trials which include patients with higher baseline plaque and inflammatory scores and subjecting them to intensive oral hygiene motivation and training sessions [22], the results of the present study probably reflect more precisely the impact of the use of different dentifrices on periodontal parameters under everyday conditions.

In conclusion, in patients suffering from mild-to-moderate chronic periodontitis, the regular use of one of both products used in the trial without professional treatment (visit 2 through 3) was accompanied by small improvements concerning plaque index, bleeding on probing and gingival index, but did not change the plaque formation rates. Despite the difference in antibacterial ingredients, i.e. AmF/SnF2 vs. microcrystalline zinc hydroxyapatite, the clinical changes in periodontal health with the regular use of each dentifrice as observed in this study were clinically indistinguishable.

## Supporting Information

S1 File PFR Protocol 203011. (PDF)

S2 File CONSORT Checklist. (PDF)
