# Supplementary material for: Impact of the Daily Use of a Microcrystal Hydroxyapatite Dentifrice on De Novo Plaque Formation and Clinical/Microbiological Parameters of Periodontal Health. A Randomized Trial
Source: PLoS One. 2016 Jul 28;11(7):e0160142. doi: 10.1371/journal.pone.0160142 (PMC4965058; doi:10.1371/journal.pone.0160142)
Supplement: S1 File — (PDF) [file pone.0160142.s001.pdf]

"Efficacy of a new carbonate/hydroxyapatite nanocrystal dentifrice on the dental plaque index and the de novo plaque formation rate in individuals suffering from gingivitis and/or periodontitis"

"PFR-Study"

BR-UM-01-2011

Version 1.0

02.03.2011

Index

**1**	**General Information**	**3**
1.1	Signatures	5
1.2	Synopsis of the Trial (Deutsch)	6
1.3	Study Activity Chart	8
**2**	**Introduction**	**9**
2.1	Potential risks of the trial	9
**3**	**Study Objectives and Purpose**	**10**
**4**	**Study Design**	**11**
4.1	Examiner Calibration	11
**5**	**Selection and Withdrawal of Subject**	**12**
5.1	Subject Inclusion Criteria	12
5.2.	Subject Exclusion Criteria	12
5.3	Subject Withdrawal Criteria	12
**6**	**Subject Registration and Randomization**	**13**
**7**	**Blinding of the Study Products and Periodontal Therapy**	**14**
7.1	Blinding of the Study Products	14
7.2	Treatment of subjects	14
7.3	Sequence of trial periods	14
**8**	**Assessment of Efficacy**	**20**
8.1	Clinical Examinations	20
8.2	Specification of Psychological Assessments	20
8.3	Microbiological Examinations	20
8.4	Laboratory Processing, Microbiological Analysis	21
**9**	**Assessment of Safety**	**22**
9.1	Definitions	22
9.2	Recording and Assessment of Adverse Events (AE)	23
9.3	Handling of Protocol Violations	23
**10**	**Statistics**	25
**11**	**Documentation, Data Management and Archiving**	**26**
**12**	**Ethics and Regulations**	**27**
**13**	**References**	**28**

1 General Information

Sponsor

Dr. Adolf Klenk
Head of R&D
Dr. Kurt Wolff Gmbh & Co KG
Johanneswerkstr. 34-36, 33609 Bielefeld, Germany
Phone: +49 521 8808 552
Fax: +49 521 8808 178
e-mail: adolf.klenk@alcina.de

Principle Investigator, Münster:

Prof. Dr. Benjamin Ehmke
University of Münster, Department of Periodontology
Waldeyerstr. 30, 48149 Münster, Germany
Phone: +49-251-8347059
Fax: +49-251-8347134
e-mail: ehmke@uni-muenster.de

Lead investigator, Würzburg

Prof. Dr. Ulrich Schlagenhauf
University of Würzburg, Department of Periodontology
Pleicherwall 2, 97070 Würzburg
Phone: +49 931-20172620
Fax: +49-931-20172680
e-mail: Schlagenhauf_U@klinik.uni-wuerzburg.de

Project Manager, Münster:

OÄ Dr med. dent. Inga Harks
University of Münster, Department of Periodontology
Waldeyerstr. 30, 48149 Münster, Germany
Phone: +49-251-8347059
Fax: +49-251-8347134
e-mail: inga.harks@uni-muenster.de

Study nurse:

Martina Gravemeier
University of Münster, Department of Periodontology
Waldeyerstr. 30, 48149 Münster, Germany
Phone: +49-251-8345175
Fax: +49-251-8347134
e-mail: mgrave@uni-muenster.de

Project Manager, Würzburg:

Dr. med. dent. Yvonne Jockel-Schneider
University of Würzburg, Department of Periodontology
Pleicherwall 2, 97070 Würzburg
Phone: +49 931-20172620
Fax: +49-931-20172680
e-mail: Jockel-Schneider_Y@klinik.uni-wuerzburg.de

Laboratory Investigator:

Dr. rer. medic. , Dipl. Biol. Karola Prior
University of Münster, Department of Periodontology
Waldeyerstr. 30, 48149 Münster, Germany
Phone: +49-251-8347044
Fax: +49-251-8347134
e-mail: karola.prior@ukmuenster.de

Clinical Investigator:

Dr. med. dent. Anna-Maria Kettner
ZÄ Marie-Christin Ohlmeier
ZA Moritz Scheidgen
University of Münster, Department of Periodontology
Waldeyerstr. 30, 48149 Münster, Germany
Phone: +49-251-8347059
Fax: +49-251-8347134
e-mail: AnnaMaria.Kettner@ukmuenster.de
Mariechristin.Ohlmeier@ukmuenster.de
Moritz.Scheidgen@ukmuenster.de

Dr. med. dent. Markus Bechtold
ZA Kai Fischer
University of Würzburg, Department of Periodontology
Pleicherwall 2, 97070 Würzburg
Phone: +49 931-20172620
Fax: +49-931-20172680
e-mail: Bechtold_M@klinik.uni-wuerzburg.de
Fischer_K@klinik.uni-wuerzburg.de

1.1 Signatures

**Sponsor**	**Dr. Adolf Klenk**	______________________<br>**Date**<br><br>______________________<br>**Signature**
**Principle Investigator Münster**	**Prof. Dr. B. Ehmke**	______________________<br>**Date**<br><br>______________________<br>**Signature**
**Lead investigator Würzburg**	**Prof. Dr. U. Schlagenhauf**	______________________<br>**Date**<br><br>______________________<br>**Signature**
**Project Manager**<br><br>**Münster**	**Dr. med. dent. I. Harks**	______________________<br>**Date**<br><br>______________________<br>**Signature**
**Würzburg**	**Dr. med. dent. Y. Jockel-Schneider**	______________________<br>**Date**<br><br>______________________<br>**Signature**
**Laboratory Investigator**	**Dr. rer. medic. , Dipl. Biol. K. Prior**	______________________<br>**Date**<br><br>______________________<br>**Signature**

1.2 Synopsis of the trial (Deutsch)

**Titel**	**„PFR Studie“ (Plaque formation rate)**
**Hauptprüfer/-prüfzentrum Münster**<br><br><br><br>**Projektkoordination**<br><br><br><br><br><br><br><br><br><br>**Prüfzentrum Würzburg**<br><br><br><br>**Koordination Würzburg**	**Prof. Dr. med. dent. Benjamin Ehmke, LKP**<br>Universitätsklinikum Münster, Poliklinik für Parodontologie, Waldeyerstr. 30, 48149 Münster, Tel.: +49-251-8347059, Fax.: +49-251-8347134, E-Mail.: ehmke@uni-muenster.de<br><br>**OÄ Dr. med. dent. Inga Harks**<br>Universitätsklinikum Münster, Poliklinik für Parodontologie, Waldeyerstr. 30, 48149 Münster, Tel.: +49-251-8347059, Fax.: +49-251-8347134, E-Mail.: inga.harks@ ukmuenster.de<br><br>**Martina Gravemeier, Studienassistentin/ DH**<br>Universitätsklinikum Münster, Poliklinik für Parodontologie, Waldeyerstr. 30, 48149 Münster, Tel.: +49-251-8345175, Fax.: +49-251-8347134, E-Mail.: mgrave@uni-muenster.de<br><br>**Prof. Dr. med. dent. Ulrich Schlagenhauf**<br>Universitätsklinikum Würzburg, Abteilung für Parodontologie, Pleicherwall 2, 97070 Würzburg, Tel: +49 931-20172620, Fax: +49-931-20172680, E-Mail: schlagenhauf@klinik.uni-wuerzburg.de<br><br>**Dr. med. dent. Yvonne Jockel-Schneider**<br>Universitätsklinkum Würzburg, Abteilung für Parodontologie, Pleicherwall 2, 97070 Würzburg, Tel: +49 931-20172620, Fax: +49-931-20172680, E-Mail: Jockel_Y@klinik.uni-wuerzburg.de
**Erkrankung**	Parodontitis / Gingivitis<br>(kontrollierte Untersuchung zur sekundären Prävention)
**Ziel der Untersuchung**	Die vorliegende Studie untersucht die folgenden Fragen: **(i)** Wie groß ist der Nutzen einer neuen Zahncreme bei der Verringerung der Plaquebildung an Zähnen und **(ii)** Verbessert die neue Zahncreme die gingivale Gesundheit **(iii)** **(iii)** Beeinflusst die Verwendung der neuen Zahncreme die mundbezogene Lebensqualität?
**Hypothese**	Die verwendete neue Zahncreme reduziert die Plaquebildungsrate bei Patienten mit Gingivitis oder leichter bis mittelschwerer Parodontitis.
**Behandlung**	Experimentelle Intervention: Mundhygieneinstruktion und mechanisches Debridement mit zusätzlicher Verwendung einer neuen Zahncreme (mind. 2x tägl. für 3 Monate).<br>Kontrollintervention: Mundhygieneinstruktion und mechanisches Debridement mit zusätzlicher Verwendung einer konventionellen Zahncreme (mind. 2x tägl. für 3 Monate).<br>Dauer der Intervention pro Patient: 3 Monate.<br>Die Kontrollintervention entspricht dem „Goldstandard“ bei der Behandlung von Gingivitis/Parodontitis.
**Risiken der Studie**	Es bestehen für Test- und Kontrollgruppe keine Risiken, die nicht auch bei der Routinetherapie bestehen. Hierzu gehören für beide Gruppen:<br>– überempfindliche Zahnhälse (temporäre Hypersensibilitäten),<br>– Nervläsionen nach zahnärztlichen, intraoralen Lokalanästhesien (temporäre oder permanente Hyposensibilität/Anästhesie),<br>– allergische Reaktionen auf Lokalanästhetika (allergischer Schock).<br>– allergische Reaktionen auf Inhaltsstoffe der verwendeten Zahncremes
**Studiendesign**	Doppelblinde, randomisierte, Placebo kontrollierte, multizentrische Untersuchung im Parallelgruppendesign (Phase IV Studie).

**Primär Endpunkt („efficacy“)**	Der Unterschied (%) in der Plaquebildungsrate.
**Sekundäre Endpunkte**	**(i)** Subjektive Wahrnehmung des Behandlungsergebnisses durch den Patienten; **(ii)** Veränderung der Menge der sub- und supragingivalen Plaque/ des Gingiva Index, **(iii)** Veränderung der Taschensondierungstiefen, **(iv)** der Blutung auf Sondierung und **(v)** der Prozentsatz der Stellen mit Attachmentgewinn (≥2 mm).
**Anzahl Prüfzentren und Patienten**	Teilnehmende Zentren sind die Poliklinik für Parodontologie des Universitätsklinkums Münster (koordinierendes Zentrum) und die Abteilung für Parodontologie des Universitätsklinikums Würzburg.<br>Screeninguntersuchung: n=140<br>Patienten die in die Studie aufgenommen werden: n=75<br>Patienten die ausgewertet werden: n=60
**Haupteinschlusskriterien**	Parodontaler Screening Index Grad I-III, max. zwei Sextant PSI Grad IV in, Patienten mit mindestens 10 Zähnen und einem Lebensalter von 18-75 Jahren. Nichtraucher
**Hauptausschlusskriterien**	Systemische Erkrankungen oder Medikamenteneinnahme die einen relevanten Einfluss auf parodontale Parameter nehmen, Schwangerschaft oder Stillperiode, obligate Antibiotikaprophylaxe bei zahnärztlichen Eingriffen.
**Untersuchungen (“visits”)**	Die Untersuchung teilt sich in die Initialphase, die Behandlungsphase und in die Nachsorgephase (follow up) ein. Insgesamt sind 6 Untersuchungs- /Behandlungstermine vorgesehen.<br>Untersuchungszeitraum: 9 Monate (first patient in through last patient out)
**Finanzielle Förderung**	Dr. Kurt Wolff GmbH & Co. KG<br>Johanneswerkstr. 34-36<br>33611 Bielefeld, Germany

1.3 Study Activity chart

	Oral hygiene Phase			Treatment		Reevaluation	
**Visit**	**1**	**2a**	**2b*****	**3a**	**3b*****	**4a**	**4b*****
	Recruitment	Baseline		Treatment*		Reev**	
**Recruitment**							
Periodontal Screening	X						
Medical Health History	X	X	X	X	X	X	X
Inclusion/exclusion criteria	X						
Study information	X						
Informed consent	X						
Registration	X						
Dental Inspection	X	X		X		X	
Randomization/balancing		X					
**Treatment activities**							
Oral hygiene instructions		X	X	X	X	X	
Supragingival debridement (full mouth)		X	X	X	X	X	X
Subgingival debridement				X	X		
Drug dispense/return		X					X
**Examinations**							
Plaque formation index			X		X		X
Plaque index		X		X		X	
Gingiva index		X		X		X	
Gingival recession		X		X		X	
Bleeding on probing		X		X		X	
Pocket probing depth		X		X		X	
Furcation involvement		X				X	
Mobility		X				X	
Clinical inspection		X	X	X	X	X	X
AE		X	X	X	X	X	X
X-rays (Zahnfilmstatus)		X					
Intraoral photography		X				X	
**Microbial samples**							
Subgingival samples		X		X		X	
Supragingival samples (buccal and lingual)		X		X		X	
Interproximal samples (mesial and distal)		X		X		X	
**Smoking**							
CO measurements	X	X		X		X	
**Questionnaires**							
OHIP		X				X	
ZUF-8		X				X	

* Within approx. 4 weeks after baseline
** Two months after treatment, ± 14 days
*** Visits a + b within 24 hours

Table1

2 Introduction

Periodontal pathogens play a well documented role in the etiology of gingivitis and periodontitis. Therapy is directed primarily towards mechanically reducing the number of pathogenic bacteria adherent to tissue. Therefore, mechanical plaque removal is the basis of most periodontal treatment regimes. For home care, different kinds of toothbrushes and dentifrices are the most frequently used oral hygiene devices (Löe 1979). Commonly, dental plaque is disrupted supragingivally directly by mechanical action of the bristles and swept away from the dental surface. The cleaning efficacy of various toothbrush designs correlates with brushing time (Huber *et al.*, 1985), and is significantly enhanced by the use of toothpaste (Davis 1980; Forward 1991). In populations without access to customary oral hygiene devices such as toothbrushes and dentifrices, up to 95% of dental surfaces exhibit visible plaque (Baelum *et al.*, 1986). In contrast, patients living in industrialized countries and practicing regular oral hygiene have on average only 40% to 60% of surfaces covered with plaque (Kalsbeek *et al.*, 2000).

It was reported, that early colonization of teeth surfaces started immediately after mechanical plaque removal. Additionally, colonization patterns were not only related to bacterial species, but also to the condition of the teeth adjacent gingiva (Saxton 1973). Therefore, a dentifrice should provide both, plaque removal on and reduction of recolonization of dental surfaces. Most dentifrices improve the cleaning efficacy due to abrasive components and furthermore reduce recolonization by the addition of anti-microbial agents, i.e. zinc-fluoride. A new attempt to reduce bacterial colonization of tooth surfaces over and above the mechanical plaque removal is the addition of nanocrystal components to dentifrices. Hydroxyapatite nanocrystals may coat the surfaces of intra-oral bacteria and therefore reduce their ability to aggregate or co-aggregate on intra-oral surfaces. This should result in improved gingival health and lower de novo plaque formation.

The hypothesis of the projected trial is that the daily use of a newly formulated dentifrice, containing carbonate/hydroxyapatite nanocrystals , in an “over the counter model”, will result in significantly lower full mouth dental plaque scores than a zinc-fluoride containing dentifrice used as a positive control.

2.1 Potential risks of the trial

There are no known risks beyond the documented risks of routine periodontal therapy and daily self performed oral hygiene with changing dentifrices. Those risks are:

- temporarily increased sensitivity of teeth (dentine hypersensitivity) to thermal, evaporative, or tactile stimuli;
- nerve damage after local anesthesia (temporary or permanent);
- allergies against the ingredients of local anesthetics;
- allergies against ingredients of the dentifrices.

3 Study Objectives and Purpose

The purpose of this study is to assess the clinical efficacy of a new dentifrice, containing carbonate/hydroxyapatite nanocrystals (BioRepair®, Kurt Wolff Pharma, Bielefeld, Germany), in subjects suffering from severe gingivitis and/or mild to moderate chronic periodontitis and receiving non-surgical periodontal therapy. Clinical efficacy will be evaluated regarding clinical and microbiological parameters and compared to the use of a zinc-fluoride containing dentifrice (Meridol®, GABA, Lörrach, Germany) serving as a positive control.

Research Questions

This three-month clinical trial is conducted to answer the following research questions:

Primary Question

1. Is there a difference in de novo plaque formation as measured by the Plaque Formation Rate Index (Axelsson 1991) between subjects using the new carbonate/hydroxylapatite nanocrystal dentifrice and those using the established zinc-fluoride containing control dentifrice?

Secondary Questions

2. Are there differences between subjects using the two dentifrices with respect to their subjective perception of therapy?
3. Are there differences between the two dentifrices with respect to the following periodontal measurements: change of full mouth plaque scores (O'Leary Plaque Control Record, O'Leary 1972), gingival index (Löe 1967), change of pocket probing depths (PPD's), and the percentage of sites with attachment gain of ≥ 2 mm?
4. Are there qualitative and quantitative changes regarding the composition of the oral microflora?

4 Study Design

The investigation is designed as a double blind, parallel group, and randomized trial over a 3-months period (primary endpoint). Secondary endpoints are not scheduled. Seventy-five subjects will be enrolled in this four-visit study; visits 1-4 are required for each subject (see Table 1). Test and control group subjects will be treated following identical therapy protocols, except for the dentifrice. Participating study centers will be the Department of Periodontology at the University of Münster and the Department of Periodontology at the University of Würzburg.

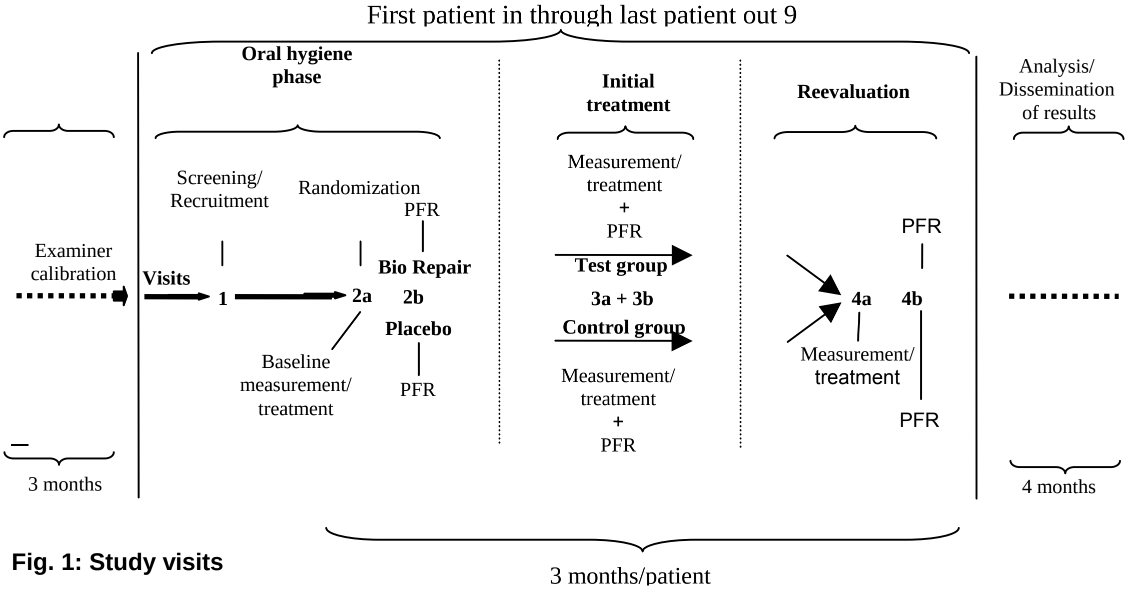

Fig. 1: Study visits

4.1 Examiner training and calibration

The blinded examiners attend a training and calibration program aimed at reviewing the objectives of the study and the study protocol. The examiners are instructed in compilation of the case report form and in optimized patient accrual and attendance. The investigator is instructed and calibrated in the measurement technique to be employed. Calibration is performed for measurements of the dichotomous plaque index systems to ensure that 95% of differences of repeated measurements are less than two standard deviations of the mean difference between two measurements (Bland and Altman 1986). For the assessment of all other clinical measurements, the examiners are trained to use standard procedures. Laboratory analyses are performed by trained laboratory technicians using evaluated standard methods.

5 Selection and Withdrawal of Subjects

5.1 Subject inclusion criteria

Subjects selected for the study must meet the following inclusion criteria:

- Periodontal screening index (PSI) I-III, PSI IV in at most two sextants, (except third molars);
- pocket probing depths (PPDs) of ≥ 4 mm at a minimum of four teeth (except third molars);
- age ≤ 18 to ≤ 75 years;
- clinical and/or radiographic signs of severe gingivitis and/or mild to moderate chronic periodontitis;
- at least 10 natural teeth in situ (except third molars);
- non-smokers (less than 3 ppm CO in exhaled air);
- no professional prophylaxis during the 1 month preceding the baseline clinical evaluation;
- willingness to participate and to be available at all times required for participation;
- willingness to abstain from using antimicrobial mouthrinses for the study duration.

5.2 Subject exclusion criteria

Subjects are excluded from the study if they:

- Have known systemic diseases that may influence the periodontal conditions, in particular Down's syndrome, known AIDS/HIV or diabetes type I or II;
- regularly take drugs that may affect the periodontal conditions, e.g. phenytoine, nifedipine, and/or anti-inflammatory drugs;
- require antibiotic treatment for dental appointments;
- are undergoing or require extensive dental or orthodontic treatment;
- are pregnant or breastfeeding;
- have rampant decay;
- any oral or extraoral piercing in or around the oral cavity with ornaments or accessory jewelry;
- currently use bleaching trays or strips;
- are dental students or dental professionals;
- work for a company that produces dental products;
- have participated in a clinical dental trial in the six months preceding the study.
- professional periodontal therapy during 6 months prior to baseline

5.3 Subject withdrawal criteria

Individual subjects discontinue the study if they:

- Avowedly offend against or are not willing to follow the protocol any more;
- do not keep the appointments.

Subjects are discharged from the study by filling in the subject accountability form. A replacement of withdrawn study subjects is not intended as long as a total of 30 subjects per group are not undershot.

Withdrawn subjects are offered the opportunity of routine periodontal therapy at the Departments of Periodontology, University of Münster and Würzburg, Germany.

6 Subject Registration and Randomization

Subjects that fulfil all the inclusion criteria, do not meet any exclusion criteria, and have signed the informed consent will be registered into the study by the centers Münster or Würzburg (visit 1). Subjects are assigned to a code number consisting of the one digit center code (1 or 2, see below) and a two digit registration number (counting backwards from 99 in every participating center). At visit 2 study subjects will be randomly assigned to the test group using the carbonate/hydroxyapatite nanocrystal dentifrice or the control group using a zinc-fluoride containing dentifrice. Patient randomization is warranted by using a randomization list (block randomization with 4 subjects per block). Designation of a study subject as a smoker/non-smoker will be based on recording carbonmonoxide concentration in exhaled air (non-smoker: less than 3 ppm CO in exhaled air; smoker: equal to or more than 3 ppm). The subjects receive the next available medication number from the center specific prepared lists (three digit medication number). This number will be fixed on the patients CRF.

Digit Center Codes:

Münster 1
Würzburg 2

Example:
Visit 1: Patients ID: 1-99
Visit 2: Next available medication number: 116

7 Blinding of the Study Products and Periodontal Therapy

7.1 Blinding of the Study Products

For elimination of measurement errors affecting one group of subjects more than the other, blinding is performed. Neither the clinical investigators nor the therapists, nor the study subjects themselves will be aware of the actual assignment to the test or the control group.

7.2 Treatment of subjects (Table 1 and Fig. 1)

At visit 1 (“Screening/Recruitment”) all subjects are informed about the projected trial. If subjects are willing to participate, they receive periodontal screening examination (PSI) and medical history is asked. At visit 2 (“Baseline”, consist of two appointments at intervals of 24 hours) within the first appointment, subjects are randomized and periodontal parameters are examined (Table 1). The blinded packages of different dentifrices are dispensed to the subjects. At the end, the subjects receive routine supragingival scaling with sonic scalers and polishing with an air powder device (glycin powder). Subjects are instructed to abstain from oral hygiene for the next 24 hours. At the end, subjects fill out the two questionnaires. At the second appointment, plaque formation rate is examined and subjects are instructed to brush with their already used toothbrush for 2 minutes at least twice per day at home.

Four weeks after “Baseline” at visit 3 (“Treatment”, consist of two appointments at intervals of 24 hours), periodontal parameters are examined and all subjects receive full mouth supra- and subgingival debridement in two sessions on two consecutive days. Debridement is performed with sonic scalers using micro tips under local anesthesia and polishing is performed with an air powder device. Subjects are instructed to abstain from oral hygiene for the next 24 hours. At the second appointment, plaque formation rate is examined, all subjects receive full mouth supra- and subgingival debridement, and are instructed to brush with their already used toothbrush for 2 minutes at least twice per day at home. Two months after “Treatment” at visit 4 (“Reevaluation”, consist of two appointments at intervals of 24 hours) periodontal parameters are examined and all subjects receive full mouth supragingival debridement in one session. Subjects are instructed to abstain from oral hygiene for the next 24 hours. At the second appointment, plaque formation rate is examined, all subjects receive full mouth supragingival debridement. Subjects return the dentifrice packages.

Over the course of the study no medication except the dentifrice or local anesthesia is administered. During the trial, subjects are not permitted to undergo advanced periodontal treatment (i.e. periodontal surgery) or to receive drugs influencing periodontal health.

7.3 Sequence of trial periods

Visit 1, Screening and Recruitment

The objective of the screening examination is to identify and recruit potential subjects for the investigation. In this preliminary examination, the periodontal screening and recording (Periodontal Screening Index [PSI]) is performed at 6 sites per tooth and the general inclusion/ exclusion criteria and medical health history are to be simultaneously verified before entry into the study. Smoking habits are determined by an objective chairside measurement of carbon monoxide concentration in exhaled air (Bedfont-Smokerlyzer®, Bedfont, UK). Information about the trial, relevant for participating, is given to the subjects. This includes:

- that the study is designed to evaluate the effects of two different dentifrices on periodontal health;
- that the subjects are randomly assigned to one type of dentifrice;
- the number of study visits, the approximate time needed to complete the visits (Visit 1: 15 + 15 min., Visit 2a/b: 60 + 15 min., Visit 3a/b: 120+15 min, Visit 4a/b: 45 +15 min.), the therapy, the clinical measurements, and all sampling procedures (overall 5 hours, 4 hours for routine therapy and one hour for additional study needs).

Only subjects signing the informed consent form approved by the Ethics Committee of the Medical Council of Unterfranken, Germany, are included in the study and are given a copy of the signed consent form. The investigators fill out a screening form (see visit 1) to document that all inclusion criteria are met. All patients are informed that generally every visit b is scheduled 24 hours after the visit a, the visit 3 is scheduled 4 weeks ±7 days after visit 2, and visit 4 is scheduled 2 months ±2 weeks after visit 3.

The baseline appointments (visit 2a+b) are scheduled and all case report forms for future visits are prepared. Subjects have to be assigned consecutive numbers consisting of a total of three digits to encode the subject and the center.

Visit 2a, “Baseline”

The objective of the baseline appointment is the complete baseline examination and measurement-taking, randomization of the study subjects, plaque sampling, oral hygiene instruction, dentifrice dispensing, and supragingival debridement and completion of the questionnaires. After checking the medical health history the examiner is performing the clinical inspection of the oral cavity.

1. Measurements

After assessment of the gingival index, except third molars, (Löe 1967), all teeth are stained with Mira 2-Ton® (Mirodent) and the full mouth plaque, except third molars, (O’Leary 1972) is examined. Clinical measurements of full mouth pocket probing depths (PPD), bleeding on probing (BOP), gingival recessions (GR), mobility, and furcation involvement are taken. All measurements, except for furcation involvement, are taken with a standard Florida Probe® handpiece. Measurements of furcation involvement are performed using a manual furcation probe (Nabers Probe). Smoking habits are determined by an objective chairside measurement of carbon monoxide concentration in exhaled air (Bedfont-Smokerlyzer®, Bedfont, UK). After completion of the clinical measurements and examinations, intraoral radiographs are taken in paralleling technique and both questionnaires (OHIP and ZUF-8) are completed. Intraoral photographs (front view) are taken and prints are stored in database.

2. Randomization / balancing and sample tooth selection

While intraoral radiographs are being taken, the subjects are assigned randomly to test or control dentifrices using pre-defined randomization tables. Four sample teeth with at least one site with PPDs of ≥ 4 mm are determined as a sample site as shown in Fig. 2 to ensure equal distribution throughout the mouth, and remain the same over the course of the study (except third molars).

Pooled subgingival plaque samples are taken from the four sample teeth for microbiological analysis. The teeth are air dried and isolated with cotton rolls. One sterile paper point is inserted for 10 seconds in each site and all paper points are removed in one transport tube containing 500 µl Ringer-Glycerin-Solution. The pooled samples are at once taken to the laboratory for analysis or stored in liquid nitrogen.

For supragingival samples, from the buccal and lingual sites samples are taken using Periotron® adapted for 10 seconds to the tooth surface to collect dental plaque from the area near the gingival margin. Interproximal, supragingival plaque samples are collected by inserting a sterile paperpoint horizontally in buccal to lingual direction for 10 seconds near the gingival margin. Both samples are pooled (buccal/lingual and mesial/distal) in a seperate transport tube containing 500 µl Ringer-Glycerin-Solution. The pooled samples are at once taken to the laboratory for analysis or stored in liquid nitrogen.

3. Oral hygiene instruction

Subjects are instructed to brush with the assigned dentifrice and with their already used toothbrush for 2 minutes at least twice per day at home. No additional instructions are given, but subjects are instructed to abstain from oral hygiene for the next 24 hours.

4. Dentifrice package dispense

The patients receive either a dentifrice containing carbonate/hydroxyapatite nanocrystals (BioRepair®, Kurt Wolff Pharma, Bielefeld, Germany), a zinc-fluoride containing dentifrice (Meridol®, GABA, Lörrach, Germany) .

5. Supragingival debridement

All subjects receive routine full mouth supragingival debridement using sonic scalers and polishing using an air powder device (glycin powder).

Visit 2b (24 hours after Visit 2a)

After checking the medical health history the examiner is performing the clinical inspection of the oral cavity. All teeth are stained with Mira 2-Ton® (Mirodent) and plaque formation rate index, except third molars, (Axelsson 1991) is examined. The Subjects receive again full mouth polishing using an air powder device (glycin powder) and are instructed to brush with their already used toothbrush for 2 minutes at least twice per day at home.

The treatment appointments (visit 3a+b) are scheduled.

Figure 2: Sample tooth selection

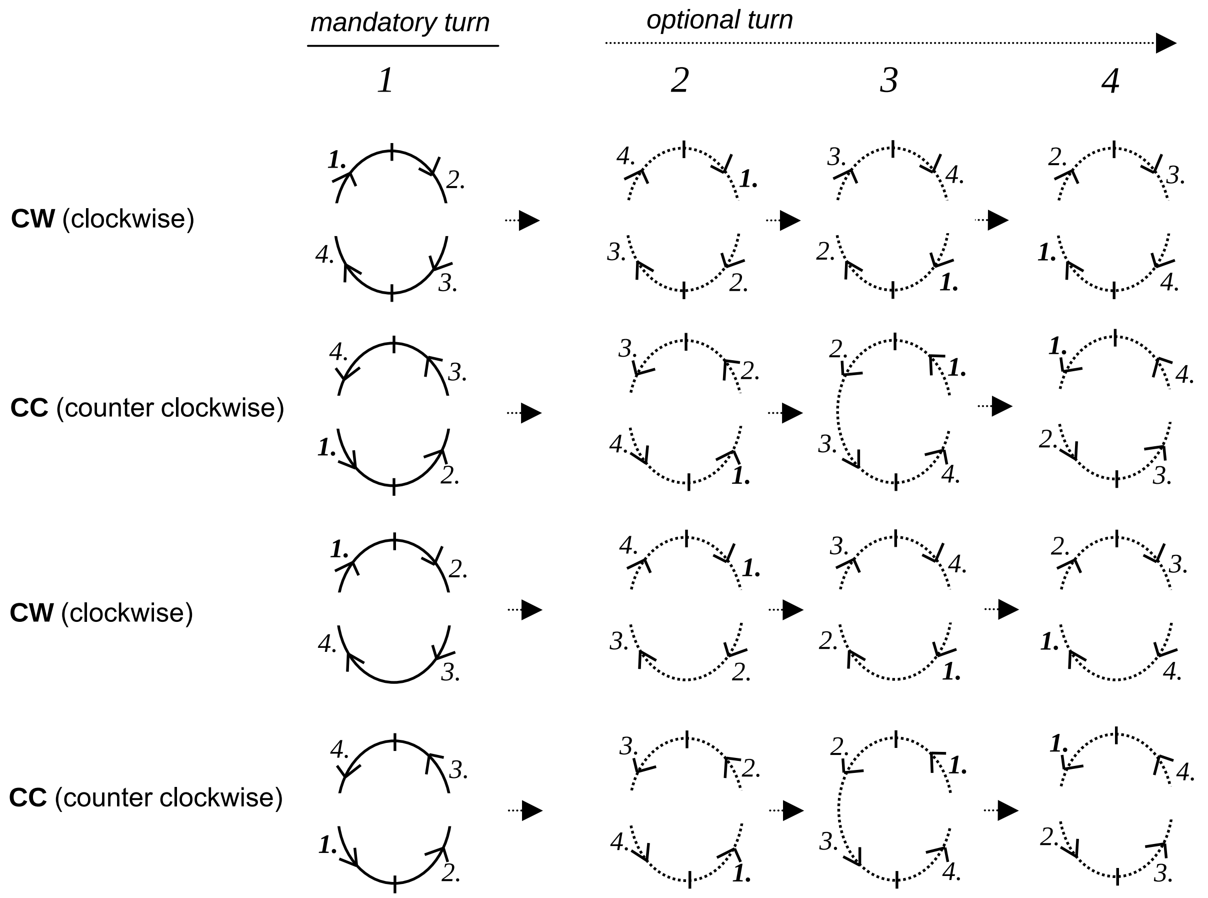

Selection process:
According to the randomization list, sample teeth are selected in a clockwise (CW) or counter clockwise (CC) direction.

Example:
For **clockwise** selection, start at the upper right quadrant from the most distal tooth going towards the mesial (CW, mandatory turn 1)

- Select the most distal tooth in this quadrant that meets the icriteria (at least one site with at least one site with PPDs of ≥ 4 mm) if one tooth is selected or none meets the criteria
- continue clockwise in the upper left quadrant and now select the most mesial tooth that meets the inclusion criteria if one tooth is selected or none meets the criteria
- continue clockwise in the lower left quadrant and select the most distal tooth that meets the inclusion criteria if one tooth is selected or none meets the criteria
- continue clockwise in the lower right quadrant and select the most mesial tooth that meets the inclusion criteria stop if one tooth in each quadrant has been selected, if less than four teeth have been selected
- continue clockwise (optional turn 2), and start in the upper left quadrant going from mesial towards distal
- continue clockwise with optional turns 3 and 4 until a total number of 4 sample teeth have been included

For **counter clockwise** proceed as indicated in the figure above

Visit 3a (4 weeks from baseline)

The objectives of this appointment are the clinical measurements and the collection of the plaque samples. After sampling and measurements, all subjects receive full mouth supra- and subgingival

debridement in two sessions on two consecutive days. After checking the medical health history the examiner is performing the clinical inspection of the oral cavity.

1. Measurements

After assessment of the gingival index (Löe 1967), all teeth are stained with Mira 2-Ton® (Mirodent) and the full mouth plaque (O'Leary 1972) is examined. Clinical measurements of full mouth pocket probing depths (PPD), bleeding on probing (BOP), and gingival recessions (GR). All measurements are taken with a standard Florida Probe® handpiece. Smoking habits are determined by an objective chairside measurement of carbon monoxide concentration in exhaled air (Bedfont-Smokerlyzer®, Bedfont, UK).

2. Microbiological samples

Plaque samples are taken as described for visit 2a.

3. Subgingival debridement

Debridement is performed with sonic scalers using micro tips under local anesthesia. Subgingival debridement is started in the upper right jaw and continued in the lower right jaw. In each case (visit 3a and 3b), full mouth polishing is performed using an air powder device. Subjects are instructed to refrain from brushing for the next 24 hours. No adjunctive antimicrobial therapy is administered.

Visit 3b (24 hours from Visit 3a)

After checking the medical health history the examiner is performing the clinical inspection of the oral cavity. All teeth are stained with Mira 2-Ton® (Mirodent) and plaque formation rate index (Axelsson 1991) is examined. At the second day, subgingival debridement is started in the upper left jaw and continued in the lower left jaw, full mouth polishing is performed using an air powder device. The subjects are instructed to brush their teeth at least twice daily.

The reevaluation appointments (visit 4a+b) are scheduled.

Visit 4 (2 months from Treatment)

The objective of the final appointment is to perform clinical measurements, dentifrice package return, and microbiological sampling. After checking the medical health history the examiner is performing the clinical inspection of the oral cavity.

1. Measurements

After assessment of the gingival index (Löe 1967), all teeth are stained with Mira 2-Ton® (Mirodent) and the full mouth plaque (O'Leary 1972) is examined. Clinical measurements of full mouth pocket probing depths (PPD), bleeding on probing (BOP), gingival recessions (GR), mobility, and furcation involvement are taken. All measurements, except for furcation involvement, are taken with a standard Florida Probe® handpiece. Measurements of furcation involvement are performed using a manual furcation probe (Nabers Probe). Smoking habits are determined by an objective chairside measurement of carbon monoxide concentration in exhaled air (Bedfont-Smokerlyzer®, Bedfont, UK). The patients completed both questionnaires (OHIP and ZUF-8). Intraoral photographs (front view) are taken and stored in database.

2. Microbiological samples

Plaque samples are collected in the same manner and from the same teeth as described for visit 2a.

3. Dentifrice package return

The participating subjects return the dentifrice package to the test center.

Visit 4b (24 hours from Visit 4a)

After checking the medical health history the examiner is performing the clinical inspection of the oral cavity. All teeth are stained with Mira 2-Ton® (Mirodent) and plaque formation rate index (Axelsson 1991) is examined. The Subjects receive again full mouth polishing using an air powder device (glycin powder).

8 Assessment of efficacy

8.1 Clinical Examinations

After assessing the plaque and the gingival index (O'Leary 1972, Löe 1967) clinical measurements of full mouth pocket probing depths (PPD), bleeding on probing (BOP), gingival recessions (GR), and furcation involvement are taken. All measurements, except for furcation involvement, are taken at six sites per tooth (mesiobuccal, mid-buccal, distobuccal, mesiolingual, mid-lingual and distolingual). Measurements of PPD, BOP, and GR are performed using a standard Florida Probe® handpiece (Florida Probe Corp., Gainsville, FL, USA). Clinical measurements are taken at baseline, after 4 weeks, and after 3 months (visits 2a, 3a, and 4a). Smoking habits are determined by an objective chairside measurement of carbon monoxide concentration in exhaled air (Bedfont-Smokerlyzer®, Bedfont, UK) at visits 1 through 4. Intraoral radiographs and photographs are taken at baseline.

8.2 Specification of Psychological Assessments

At visits 2a and 4a patients are asked to complete the Oral Health Impact Profile – German Version (OHIP-G 49) and the German version of the Client Satisfaction Questionnaire (ZUF-8) as a standardized instrument used to measure health outcome.

Oral Health Impact Profile – German Version (OHIP-G 49)

The original English version of the questionnaire was developed by Slade and Spencer (1994). The German translation and validation of the instrument was performed by John *et al.* (2002). The OHIP-G 49 contains 49 items that address specific problems of oral health, e.g., "problems with chewing of food" or "pain in the gums". Each item is assessed on a five-point scale (0: *never* to 4: *very often*). A sum score of all of the items is calculated as an indicator for current oral health. The completion of the questionnaire takes about ten minutes.

ZUF-8

The patients´ satisfaction with the treatment is evaluated by means of the *Fragebogen zur Patientenzufriedenheit* (ZUF-8, Schmidt *et al.* 1989) which represents the German version of the *Client Satisfaction Questionnaire* (CQS, Attkisson & Zwick 1982).

8.3 Microbiological Examination

For microbiological analysis, pooled subgingival plaque samples are taken from four different teeth with at least one site with PPDs of ≥ 4 mm from each study subject. The pooled subgingival plaque sample is collected in a transport tube containing 500 µl Ringer-Glycerin-Solution. The pooled samples are at once taken to the laboratory for analysis or stored in liquid nitrogen.

Supragingival plaque is collected from four sites per sample tooth. From the buccal and lingual sites samples are taken using Periotron© adapted to the tooth surface to collect dental plaque from the area near the gingival margin. Interproximal, supragingival plaque samples are collected by inserting a sterile paperpoint horizontally in buccal to lingual direction near the gingival margin. Samples from buccal/lingual and from interproximal sites are pooled respectively (two pooled samples from each

subject per visit 2a, 3a, 4a) in a seperate transport tube containing 500 µl Ringer-Glycerin-Solution. The pooled samples are at once taken to the laboratory for analysis or stored in liquid nitrogen.
Sample teeth are randomly selected at baseline as shown in Fig. 2 to assure equal distribution throughout the mouth, and remain the same over the course of the study. The whole bacterial counts are quantified by culture at study visits 2a, 3a, and 4a.

8.4 Laboratory Processing, Microbiological Analysis

After subgingival plaque sampling, the samples are taken immediately in the laboratory for further processing or stored in liquid nitrogen until the study is finished. After finishing the trial they will be carried to Münster. For transport they are stored in carbon dioxide snow and are stored in liquid nitrogen in the laboratory in Münster again. The pooled samples are sonicated for 10 seconds (Sonotex RK 82, Bandelin Electronic AG, Berlin, Germany) and diluted in tenfold steps. 0.1 ml of the undiluted suspension and 0.1 ml aliquots of the dilutions are spread on different culture media. In parallel supragingival plaque samples are spread on CDC agar and are stored in an aerobe atmosphere at 37 degree above zero overnight.
For quantitative enumeration the undiluted and diluted suspensions are spread on non-selective blood agar (CDC agar) plates containing 5% defibrinated sheep blood supplemented with 5mg/l hemin (Merck, Darmstadt, Germany), 1 mg/l vitamin K_1 (Kleinfelder *et al.*, 2000), and 10 mg/l N-acetylmuramine acid (NAM). NAM is supplemented for cultivation of *T. forsythensis*. The plates are incubated in an atmosphere containing N_2 (85%), H_2 (10%), and CO_2 (5%) for 7 days. Total cultivable counts are assessed for each of the plaque samples. The evaluation of pooled plaque samples on CDC agar and the total cultivable counts are reported quantitatively as colony forming units (CFU/ml). In further investigation the present of specific periodontal pathogens will be evaluated.

9 Assessment of safety

To assess the safety of the investigational medicinal products all adverse events occurring during the trial period will be assessed, recorded, evaluated and reported according to national regulations.
Parameters for safety are the absence of factitious, iatrogenic and accidental traumatic lesions of periodontal tissues. Any kind of serious chemical or physical gum damage that may be related to the dispensed dentifrice leads to withdrawal of the subject. A visual inspection of the integrity of the periodontal and adjacent tissues is performed at every visit. Furthermore, in the case of tissue damage all necessary therapy is initiated to avert any irreparable injury.

9.1 Definitions

Adverse event (AE)

Any untoward medical occurrence in a patient or clinical trial subject administered a medicinal product and which does not necessarily have a causal relationship with this treatment.
Comment: An adverse event can therefore be any unfavourable and unintended sign (including an abnormal laboratory finding), symptom, or disease temporally associated with the use of an investigational medicinal product, whether or not considered related to the investigational medicinal product.

Adverse reaction (AR)

All untoward and unintended responses to an investigational medicinal product related to any dose administered.
Comment: All adverse events judged by either the reporting investigator or the sponsor as having a reasonable causal relationship to a medicinal product qualify as adverse reactions. The expression reasonable causal relationship means to convey in general that there is evidence or argument to suggest a causal relationship.

Unexpected adverse reaction (UAR)

An adverse reaction, the nature, or severity of which is not consistent with the applicable product information (e.g. investigator's brochure for an unapproved investigational product or summary of product characteristics for an authorized product).
Comments: When the outcome of the adverse reaction is not consistent with the applicable product information this adverse reaction should be considered as unexpected.

Serious adverse event or serious adverse reaction (SAE or SAR)

Any untoward medical occurrence or effect that at any dose

- results in death,
- is life-threatening,
- requires hospitalization or prolongation of existing inpatients´ hospitalization,
- results in persistent or significant disability or incapacity,
- causes a congenital anomaly or a birth defect.

- Comments:
- Life-threatening in the definition of a serious adverse event or serious adverse reaction refers to an event in which the subject was at risk of death at the time of event; it does not refer to an event which hypothetically might have caused death if it were more severe.
- Medical judgment should be exercised in deciding whether an adverse event/reaction is serious in other situations. Important adverse events/ reactions that are not immediately life-threatening or do not result in death or hospitalization but may jeopardize the subject or may require intervention to prevent one of the other outcomes listed in the definition above, should also be considered serious.

Suspected unexpected serious adverse reaction (SUSAR)

A suspected serious adverse reaction that has been judged to be unexpected.

9.2 Recording and Assessment of Adverse Events (AE)

All adverse events occurring during the trial period have to be documented in the AE-form of the CRF including assessment of severity (mild: no influence on daily activities, moderate: daily activities impeded, and severe: daily activities or work not possible) and an appraisement of seriousness and causality. Additionally, it will be discriminated between expected or unexpected adverse events. Where possible, a diagnosis rather than a list of symptoms should be given. All adverse events shall be followed up until symptoms disappear. The participating centers will report all AE’s to the project manager at the coordinating center Münster. A summary of AE’s will be given to the sponsor in the final report of the study.

9.3 Handling of Protocol Violations

Planned general changes in the protocol approved by the ethics committee, i.e., protocol deviations and protocol exceptions, will be submitted as formal protocol amendments or protocol exceptions to the ethics committee and must be approved prior to initiation or implementation of the change. A list of major and minor protocol violation definitions and procedures for reporting and handling them is implemented in the protocol. A protocol violation is any protocol deviation that is not approved by the ethics committee prior to the initiation.

Major violations:

a) Failure to obtain informed consent, i.e., there is no documentation of informed consent or informed consent obtained after initiation of study procedures.
b) Enrollment of a subject who did not meet inclusion or exclusion criteria.
c) Failure to perform required measurements and, therefore, may be affect subject safety or data integrity (i.e., baseline measurements of clinical parameters).
d) Drug medication dispensing or dosing error.

Minor violations:

a) Missing of signed and dated original consent form (i.e., only a photocopy available).

b) Inappropriate documentation of informed consent (missing subject/investigator signature, someone other than the subject dated the consent form, use of invalid consent form).
c) Failure to follow the approved study procedures that, in the opinion of the coordinating investigator, does not affect subject safety or data integrity:
   - Study procedure conducted out of sequence
   - Omitting an approved portion of the protocol
   - Failure to take a required sample for lab testing
   - Study visit conducted outside of required timeframe
d) Failure of subject to return study medication and medication diary.
e) Over-enrollment.
f) Enrollment of subjects after study expired.

Reporting and handling of protocol violations

Protocol violations may be reported either (i) as a self-report of the investigator or other clinical staff involved in the trial at the participating centers, (ii) by the monitor or audit team or (iii) by patients. All investigators are obliged to immediately report major and minor violations coming to their knowledge to the coordinating investigator. All major protocol violations will be discussed within the principal investigators of Münster and Würzburg and decided on individually basis. Minor protocol violations will be handled by the coordinating investigator. The coordinating investigator may decide to involve the principal investigators in the decision about these violations.

10 Statistical analysis

For both, primary and secondary outcome parameters, the two sampled *t*-test is used to test differences between the groups.

Power analyses will use data from a pilot study.

11 Documentation, Data Management and Archiving

Subject Identification List

All patient associated data are captured in a pseudonym manner. Every participating subject is distinctively assigned to a registration number consisting of one digit center code and a two digit registration code. This number is assigned at visit one. A confidential subject identification list is stored in the examiner's folder ("Prüfarztordner") existing in every participating center. In this list the registration number is connected with the patient's name.

Data Handling and Record Keeping

The study nurse provides for each subject a folder containing all case report forms (clinical and laboratory) and copies of all forms signed by the subjects during the study period. Radiographs are also to be attached to the subject's folder. The case report form is to be filled in at the time of data collection and to be signed after completion by the investigator or by the laboratory personnel responsible. The investigator and laboratory personnel are required to return the case report forms after completion of the measurements or analysis. The study nurse copies completed case report forms and originals, and copies are stored at different places. After examinations are completed, data that are entered directly in a microcomputer (i.e. all Florida Probe® measurements) undergo daily backup on transportable media and are stored at different places. Additionally, all Florida Probe® data are also saved as printed hard copies in the patient's folder. All data are entered continuously in a microcomputer and are checked by a second person for entry errors.

Storage of Trial Documents

The trial master file containing all study related documents will be stored, according to the national requirement for at least 15 years after completion of the final report at the coordinating center. Additionally, copies of the forms and the investigator site file are stored for the same period at the corresponding study centers.

12 Ethics

This study protocol and the informed consent form are to be approved by the Ethics Committee of the MedicalCouncil of Unterfranken, Germany

The trial will be conducted in accordance with the Declaration of Helsinki (2008 Seoul), the Principles of Good Clinical Practice and the applicable German Law.

Patient Insurance

For all subjects a patient insurance will be contracted.

Study Information and Informed Consent

Prior to registration into the trial, every subject willing to participate in the trial is informed by the clinical investigator at the participating center about the character, the impact, as well as the risks and benefits of the trial. Additionally, subjects get informed that they are entitled to quit participation at all time without drawbacks concerning further periodontal therapy at the respective Department of Periodontology. In accordance with the MPG, patients are informed that disease associated data will be saved pseudonymously and will be used for scientific publications. This is documented by an intelligible study information and informed consent document (one document) signed by the patient. After signing the patient get a copy of this document (for study information and informed consent see the appendix).

Financing

The trial is exclusively funded by Dr. Kurt Wolff GmbH & Co. KG, Bielefeld, Germany.

Compliance with the Trial Protocol

All procedures concerning the present trial have to be executed conform to the definitions and instructions as given in this protocol. Every protocol deviation by the investigator concerning the scheduled measurements and treatments or scheduled the time flow has to be documented and justified (e.g. in case of emergency).

The advances in the ongoing trial, especially the scheduled recruitment rates of the participating centers, are documented in every participating center and transmitted to the coordinating investigator by a monthly report.

13 Reference List

Attkisson CC, Zwick R (1982) The Client Satisfaction Questionnaire. Psychometric properties and correlations with service utilization and psychotherapy outcome. Eval Program Plann 5:233-237.

Axelsson P (1991)
A four-point scale for selection of caries risk patients, based on salivary *S. mutans* levels and plaque formation rate index. Cambridge University press, 1991. *Risk markers for oral diseases*, Vol. I, Caries, pp. 158-170.

Bland MJ and Altman DG (1986).
Statistical methods for assessing agreement between two methods of clinical measurement. *The Lancet* 8:307-310.

Baelum V, Fejerskov O, and Karring T (1986).
Oral hygiene, gingivitis and periodontal breakdown in adult Tanzanians. *J Periodontal Res* 21:221-232.

Davis WB (1980).
Cleaning and polishing of teeth by brushing. *Community Dent Oral Epidemiol* 8:237-243.

Forward GC (1991).
Role of toothpastes in the cleaning of teeth. *Int Dent J* 41:164-170.

Huber B, Rueger K, and Hefti A (1985).
[The effect of the duration of toothbrushing on plaque reduction]. *Schweiz Monatsschr Zahnmed* 95:985-992.

John MT, Patrick DL, Slade GD (2002) The German Version of the Oral Health Impact Profile – Translation and Psychometric Properties. Eur J Oral Sci 110:425-433

Kalsbeek H, Truin GJ, Poorterman JH, van Rossum GM, van Rijkom HM, and Verrips GH (2000).
Trends in periodontal status and oral hygiene habits in Dutch adults between 1983 and 1995. *Community Dent Oral Epidemiol* 28:112-118.

Kleinfelder JW, Muller RF, and Lange DE (2000).
Bacterial susceptibility to amoxicillin and potassium clavulanate in advanced periodontitis patients not responding to mechanical therapy. *J Clin Periodontol* 27:846-853.

Löe H (1979).
Mechanical and chemical control of dental plaque. *J Clin Periodontol* 6:32-36.

Löe H (1967).
The gingival index, the plaque index and the retention index systems. *J Periodontol* 38:610-616.

O'Leary TJ (1972).
The plaque control record. *J Periodontol* 43:38-42.

Saxton CA (1975)
Scanning electron microscope study of the formation of dental plaque. *Caries Research* 7:102-109.

Schmidt J, Lamprecht F, Wittmann WW (1989) Zufriedenheit mit der stationären Versorgung. Entwicklung eines Fragebogens und erste Validitätsuntersuchungen. Psychother med Psychol 39:248-255.

Slade GD, Spencer AJ (1994) Development and Evaluation of the Oral Health Impact Profile. Community Dent Health 11:3-11.
